# Alan Lee, FRCPsych

**DOI:** 10.1192/bjb.2022.57

**Published:** 2023-02

**Authors:** Conor Duggan

Consultant in general psychiatry (retired), Nottinghamshire Healthcare NHS Trust, Nottingham, UK


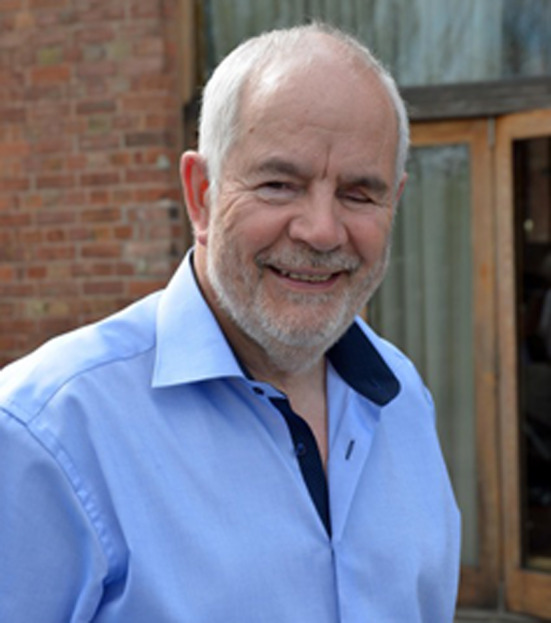


Alan Lee, who died suddenly on 23 June 2022, aged 74, was an influential contributor to knowledge about the long-term prognosis of depressive disorders. In 1988, with Robin Murray, he published a follow-up study of patients with this diagnosis made 15 years earlier at the Maudsley Hospital, London. It had previously been thought that depressive disorder was a disorder with a generally benign prognosis. Lee and Murray's study showed that fewer than one-fifth of the 79 survivors had remained well and over one-third of the series suffered unnatural death or severe chronic distress and disability.^1^ Together with the results from a similar study carried out at about the same time in Sydney, Australia, these findings made it clear that, contrary to previous belief, people who suffered depressive disorders usually needed long-term care. His practice therefore anticipated the personalised care approach long before it became fashionable and this was greatly welcomed by his patients. Subsequently, as an NHS consultant in general psychiatry, his capacity to integrate current evidence critically and thoughtfully with the unique circumstances of the individual made him an exceptional clinician. He retained an active interest in research and succeeded Andrew Sims, as the second editor of *Advances in Psychiatric Treatment*. He also acted as assistant editor to Sidney Crown, the book editor for the *British Journal of Psychiatry*.

Alan Lee was born in Ashton-under-Lyne, Lancashire (now in Greater Manchester), the only child of Kathy (née Rolls) and Joe Lee, who worked in the chemical industry. From Manchester Grammar School, he won a scholarship to Christ's College, Cambridge, to study mathematics. Influenced by his tutor Mike Brierley, who later became a psychoanalyst and a notable captain of the England cricket team, after a year he switched to philosophy and psychology. After leaving Cambridge, he became an assistant probation officer in County Durham, where his experience at a local psychiatric hospital was a key influence on his final choice of career.

He obtained a place to study medicine at the University of Newcastle Medical School and, after qualification, worked as a junior doctor in Newcastle and Southampton, before going on to train in psychiatry at the Maudsley Hospital. From the Maudsley, in 1987 he was appointed a consultant in general psychiatry in Nottingham. Subsequently, his entire consultant career was spent providing psychiatric care to the Broxtowe district in Nottingham. He was an outstanding and inspirational teacher and had significant influence on the careers of junior psychiatrists both at the Maudsley and subsequently in Nottingham. As well as his NHS work, he was an unpaid adviser to Nottingham Samaritans and Nottingham Relate.

Alan was blessed with a wry and self-deprecating humour which enthralled his many friends and acquaintances. He was ably supported by his wife Helen – a psychiatric nurse and psychotherapist – whom he met as a trainee, their marriage taking place at the Bethlem Royal Hospital. They enjoyed a happy life together and travelled extensively after his retirement. Alan was a cricket enthusiast and was a regular attender at his beloved Trent Bridge. Perhaps his greatest accolade is that he was the psychiatrist that other psychiatrists might choose when they or their family members suffered from mental illness – a true psychiatrists’ psychiatrist.
